# Estimating the government health-care costs of treating pesticide poisoned and pesticide self-poisoned patients in Sri Lanka

**DOI:** 10.1080/16549716.2019.1692616

**Published:** 2019-11-28

**Authors:** Hannah Ahrensberg, Lizell B. Madsen, Melissa Pearson, Manjula Weerasinghe, Michael Eddleston, Shaluka Jayamanne, Kristian S. Hansen, Vindya Ariyarathna, Sandamali Rajapaksha, Flemming Konradsen

**Affiliations:** a Section of Global Health, Department of Public Health, University of Copenhagen, Copenhagen, Denmark; b South Asian Clinical Toxicology Research Collaboration (SACTRC), Faculty of Medicine, University of Peradeniya, Peradeniya, Sri Lanka; c Pharmacology, Toxicology & Therapeutics, University/BHF Centre for Cardiovascular Science, University of Edinburgh, Edinburgh, UK; d Department of Community Medicine, Faculty of Medicine & Allied Sciences, Rajarata University of Sri Lanka, Anuradhapura, Sri Lanka; e Faculty of Medicine, University of Kelaniya, Kelaniya, Sri Lanka; f Department of Health Services Research, Department of Public Health, University of Copenhagen, Copenhagen, Denmark

**Keywords:** Cost analysis, suicide and self-harm, pesticide self-poisoning, treatment costs, government costs, lower-middle income country, Sri Lanka

## Abstract

**Background**: Pesticide self-poisoning as a method of suicide is a major global health problem.

**Objectives**: To estimate the cost and per patient cost of treating pesticide self-poisoning at different hospital levels in a Sri Lankan district, and to examine the distribution of cost components. Another objective was to investigate changes in total cost of treatment of pesticide poisoning for all causes at different administrative levels in Sri Lanka in 2005 and 2015.

**Methods**: The economic framework was a costing analysis, adopting a government perspective. Cost data were collected prospectively over a 4-month period in 2016 for patients admitted for pesticide self-poisoning to six hospitals in the Anuradhapura District. Assumption-based scenario analyses were run to determine changes in total pesticide poisoning treatment costs.

**Results**: We included 67 self-poisoned patients in the study. The total cost of treatment was US$ 5,714 at an average treatment cost of US$ 85.3 (9.7–286.6) per patient (across all hospital levels). Hospital costs constituted 67% of the total cost for treating self-poisoning cases and patient-specific costs accounted for 29%. Direct cost of patient hospital transfer constituted the smallest share of costs (4%) but accounted for almost half of the total costs at primary level. The estimated total cost of treating all causes of pesticide poisoning in Sri Lanka was US$ 2.5 million or 0.19% of the total government health expenditure (GHE) in 2015.

**Conclusion**: Our findings indicate that the average per patient cost of pesticide self-poisoning treatment has increased while the total cost of pesticide poisoning treatment as a percentage of the total GHE in Sri Lanka has declined over the past decade. A continuous focus on banning the most hazardous pesticides available would likely further drive down the cost of pesticide self-poisoning and pesticide poisoning to the government.

## Background

In 2016, the World Health Organisation (WHO) estimated that 793,000 people committed suicide worldwide, equalling an annual age-standardized suicide rate of 10.5 per 100,000 population globally [1]. The majority of these deaths occurred in low- and middle-income countries in Asia, including Sri Lanka with an age-standardized suicide rate of 17.1 per 100,000 in 2012 [2].

Pesticide self-poisoning is one of the most commonly used methods of suicide worldwide, and a systematic review estimated that suicide by pesticide-ingestion in 2010–2014 accounted for approximately 14% of the annual suicides [3]. The easy accessibility to pesticides in rural farming communities and the high toxicity of pesticides sold contribute towards the high burden of deaths from pesticide self-poisonings in low- and middle-income countries in Asia [4–7].

Sri Lanka differs from many other Asian countries, as it has free universal health-care coverage financed by the government. Sri Lanka has a comprehensive network of hospitals providing both preventive and curative health-care services to all [8,9]. The direct and indirect cost of pesticide self-poisoning are considerable and include the economic costs of emergency services and intensive care, loss of income during hospitalisation and recovery, and social stigma to a person and family [10]. Costing studies are an important tool for decision-makers as they provide information on the cost of illness, as well as yield important management and policy information concerning relevant cost-savings in the overall treatment of pesticide self-poisoning patients. Two cost of illness studies have previously been conducted in South Asia to determine the cost of treating pesticide self-poisoning from a governmental perspective. Wickramasinghe et al. [11] estimated the average cost of treating pesticide self-poisoning at primary and secondary hospital level in Sri Lanka to be US$ 4.5 and US$ 60.1, respectively, in 2005 (adjusted for inflation to US$ (2015)). Verma et al. [12] found that the average cost of treating self-poisoning with organophosphate in Bangladesh was US$ 218.1, split 80/20 between patient (US$ 174.4) and government (US$ 43.6) (adjusted for inflation).

The first aim of the study was to estimate the cost of treating pesticide self-poisoning cases in the Anuradhapura District, Sri Lanka, at different levels of hospital services, and to examine the distribution of different cost components to the total costs of treatment. The hospital study was part of an existing community-based cluster randomised trial (RCT) on safe storage of pesticides taking place in the district. The Wickramasinghe et al. study [11] had taken place in the same district years earlier which provided an opportunity to compare changes in average per patient treatment cost of pesticide self-poisoning over the past 10 years. Another objective of the study was to use the cost estimates obtained from the present hospital study and the Wickramasinghe study to investigate changes in the total cost of treatment of all causes of pesticide poisoning cases at different administrative levels (district, provincial, and national) in Sri Lanka over the past decade (2005/2015) through scenario analyses.

## Methods

### Study area and frame

The study took place in the Anuradhapura District, located in the North Central Province (NCP) of Sri Lanka, and is part of a large community-based cluster randomised trial investigating the effect of safe storage on the mortality of pesticide self-poisoning in Sri Lanka [13,14].

In 2015, the district had 4,064 hospital beds divided among 34 hospitals corresponding to 4.6 beds per 1,000 people. That year, 1,796 cases of pesticide poisoning (including 49 fatal cases and for all causes of pesticide poisonings) were registered at the hospitals in the Anuradhapura District. Registered cases of pesticide poisoning at the provincial and national levels were 2,628 and 15,778, respectively, of which 64 and 374 cases, respectively, were fatal [15].

### Hospital and patient sampling

Data pertaining to the cost of treating people who intentionally self-poisoned with pesticides were collected prospectively from six different hospitals in the Anuradhapura District in the period March to July 2016.

Cost data were collected from the Anuradhapura Teaching Hospital (THA), Thambuttegama Base Hospital (type B), Thalawa Divisional Hospital, Galnewa Divisional Hospital, Eppawala Divisional Hospital, and Rajanganaya Tract 11 Divisional Hospital. Divisional hospitals, base hospital, and THA are referred to as primary, secondary, and tertiary levels, respectively, in the following. An overview of levels of health-care provided at different types of hospitals in Sri Lanka is presented in Table 1.

**Table 1.** Health-care levels and types of government hospitals in Sri Lanka [16,17].

Level of health-care	Characteristics	Type of hospital	Notes
Primary health-care	Provides non-specialist inpatient and outpatient care	Primary medical care units	
		Divisional hospital	Type A, B, C
Secondary health-care	Provides health-care in different specialities^a^	Base hospital– Type A– Type B	Type B hospitals have fewer basic specialities than type A
Tertiary health-care	Provides specialised health-care in four main specialities	District General hospital (DGH)Provincial General hospital (PGH)	DGH health-care also includes some sub specialitiesPGH has more facilities than DGH
		Teaching hospital	Provides health-care in the main sub specialities, and is used for teaching purposes
		National hospital	Main hospital in Sri Lanka, also provides health-care in many sub specialities.

^a^Specialities include medicine, surgery, paediatric, obstetrics, and gynaecology.

In the present study, one case had been registered as self-poisoning with the highly hazardous pesticide, organochlorine. Poisoning with this pesticide group is unlikely as it has been banned in Sri Lanka since 1998 [18]. Furthermore, the poison type could afterwards not be validated in the medical records and the RCT dataset and was therefore not included in the final study population of 67 patients.

### Costing methodology

The economic framework is a costing analysis, which adopted a government perspective in estimating the cost of treating pesticide self-poisoning patients. The cost is exclusively treatment costs borne by the government and cost to the patient or society is therefore not included. The costing analysis included the following cost items: consumables, transfer, personnel, capital, and overhead. Unit costs and prices were obtained from official statistics, the health facilities and the Medical Supply Division of the Ministry of Health and the Provincial Department of Health. When reliable data were not available, case regional estimates on unit costs were obtained from the WHO-CHOICE database, and through personal communication with researchers in the field. Table 2 provides an overview of data types and sources.

**Table 2.** Input parameters collected to estimate the cost of treating pesticide self-poisoning in Anuradhapura District, Sri Lanka.

Input parameters	Baseline estimate	Data sources
Patient-specific cost		
- Medical prescriptions, medical devices, laboratory tests	Varies according to poison type and hospital level	Present study
Hospital cost at primary and secondary level		
- Personnel costs	Varies according to type	Present study
- Capital costs, incl. medical equipment and ambulances	Varies according to type	Present study
- Operational cost of capital cost, incl. buildings	5% of total capital cost	[19]
- Overhead	Varies according to type	Present study
- Consumables	Varies according to type	Present study
- Buildings		
◦ Construction price	LKR 5,000/6,500 per m^2^	Present study
◦ Lifetime of building	60 years	[20,21]
Hospital cost at tertiary level		
- Cost per bed day	US$ 77.65	[22]
Transfer cost (fuel cost)	US$ 1.4 per litre	Present study

All costs in the study are expressed in 2015 US dollars, using the gross domestic product deflator. Furthermore, the recommended discount rate of 3% was applied to all costs [19,20].

### Costs

The costs of treating pesticide self-poisoning cases were calculated as the sum of patient-specific treatment costs by poison type and the hospital costs of admittance, i.e. the cost per bed day by hospital level (primary, secondary, and tertiary).

#### Patient-specific treatment costs by poison type

A field assistant (pharmacy graduate) was stationed at the six hospitals at different times over the 4-month study period. The field assistant systematically registered all unit cost inputs (consumables) administered to the patient at the different hospitals until discharge. Patient treatment costs included: medical prescriptions, medical devices, and laboratory tests. Fuel costs were used as a proxy for the cost of transferring a patient from one hospital level to the next. Costs were calculated as the sum of the number of units consumed multiplied by unit cost.

#### Hospital costs

The average per bed day unit cost represents the ‘hotel’ components of a stay at a specific hospital level and includes capital, overhead and personnel costs, but excludes specific costs of the individual patient, e.g. medication and laboratory tests. The capital and operational costs for primary and secondary hospitals were calculated by collecting and summing all annual operational costs of the respective hospitals as well as the annuitized value of capital items, including buildings. The value of the buildings was calculated as the construction price of the building. Annuitisation was calculated under the assumption that the lifetime of buildings is 60 years [21,22]. It was not possible to collect sufficient data from THA and a unit cost of US$ 77.65 per bed day for tertiary hospitals in Sri Lanka was obtained from the WHO (adjusted for inflation) [23].

### Sensitivity analyses of hospital study

One-way sensitivity analyses were undertaken to assess whether parameter uncertainties impacted on the results of the analysis. Individual input parameters’ values were varied across a range of 1–100% while holding all other parameters constant to assess how these changes affected the results [19]. To investigate discounting scenarios other than the 3% rate, sensitivity analyses were run with 0% and 6% discount rates.

### Scenario analyses of changes in the total treatment cost of pesticide poisoning in Sri Lanka

In order to estimate the total cost of treating pesticide poisoning at the district, provincial and national level, our cost estimates were extrapolated to all the pesticide cases registered in Anuradhapura District, NCP and Sri Lanka (1,796; 2,564 and 15,404, respectively), in 2015 [15]. For these scenarios, the cause of poisoning was not available for the registered cases and the cost of treatment therefore pertain to all causes of pesticide poisoning cases, including occupational, accidental, and self-poisoned cases. Our findings were extrapolated to cases at different hospital levels using a ‘distribution key’ of pesticide cases by poisoning type obtained from a large, community-based, randomised trial conducted in the Anuradhapura District [14] (data not included in this paper). It was assumed that (a) all pesticide poisoning cases received medical attention, (b) none of the cases needed admission to intensive care units (ICU), (c) the cost of treating an accidental or occupational case of poisoning equalled the cost of treating a case of pesticide self-poisoning as estimated in this study, and (d) the number of pesticide cases registered by the Regional Director of Health Services Division excluded transfer cases.

In addition to the base-case scenario, several scenarios using different cost estimates were run to assess and compare changes in the total cost of pesticide treatment at the district, provincial, and national levels in the years 2005 and 2015. Cost estimates and data obtained from Wickramasinghe et al. were used to calculate the average per patient cost of pesticide self-poisoning treatment in 2005 (US$ 48.2 (4.5–60.1) (adjusted for inflation to US$ (2015))) and to estimate the total cost of treating pesticide cases in the Anuradhapura District, NCP and Sri Lanka (1,891; 2,635, and 16,910, respectively) in 2005 [11,24]. It was assumed that the number of pesticide cases registered by the Regional Director of Health Services Division for 2005 did not include treatment of transfer cases.

Key parameters for each scenario were:

- Scenario 1: Registered cases were grouped into two different pesticide categories (organophosphate/carbamate cases, and other pesticide cases) using a ‘distribution key’; the cost of transfer was excluded; and an average cost estimate obtained from this study was used for each pesticide category across all hospital levels.
- Scenario 2: Registered cases were grouped into two different pesticide categories (organophosphate/carbamate cases, and other pesticide cases) using a ‘distribution key’; the cost of transfer was included; and a cost estimate obtained from this study was used for each pesticide category by hospital level.
- Scenario 3: Registered cases were grouped into three different pesticide categories (organophosphate, carbamate, and other pesticides) using a ‘distribution key’; the cost of transfer was included; and a cost estimate obtained from this study was used for each pesticide category by hospital level.

## Results

### Patient characteristics

The study was conducted over a 4-month period in 2016, and 67 pesticide self-poisoning patients were registered at the six different hospitals. Almost 60% (n = 39) of the patients were transferred to the THA, while one patient was transferred to the District General Hospital Chilaw (DGH Chilaw) in Puttalam District for further treatment. All treatment costs incurred at DGH Chilaw were excluded. Transfer and treatment costs incurred at hospitals in the Anuradhapura District were included in the hospital study, including transfer costs out of the district. Twenty-five patients were registered at the primary hospital level, 28 patients at the secondary level, and 14 patients at the tertiary level. None of the registered cases in the study was severe enough to require admission to ICU. There were more men than women admitted for pesticide self-poisoning. The majority of patients were admitted for treatment of insecticide poisoning (72.1%), of which the most common class of pesticides ingested was organophosphate (30.9%). The average length of stay of hospital admittance was 26 h and 39 min for each patient (see Table 3 for details). An overview of the different pesticides used to self-poison identified in the study and their associated chemical group, active compound, and toxicity can be found in the Supplementary Section.

**Table 3.** Characteristics of pesticide self-poisoned patients admitted to six hospitals in Anuradhapura District, Sri Lanka, from 1 March to 31 July 2016.

Variable		*n* (%)
Patients		67 (100)
Mean age (SD)		36.15 (15.6)
Gender		
- Female		27 (40.3)
- Male		40 (59.7)
Poison type	Chemical group	
Insecticide		48 (71.6)
	- Carbamate	16 (23.9)
	- Organophosphate	21 (31.3)
	- Other	11 (16.4)
Herbicide		19 (28.4)
Average length of stay per poison type (h)		
Insecticide		31 h 38 min
	- Carbamate	25 h 34 min
	- Organophosphate	23 h 14 min
	- Other	56 h 27 min
Herbicide		15 h 28 min
Total length of stay in average (h)		27 h 2 min
Patients transferred		40 (58.8)

### Total cost and cost profile

The total cost of treating the 67 patients amounted to US$ 5,714, equalling an average cost of US$ 85.3 (9.7–286.6) per treated patient. The total cost of patient-specific treatment amounted to US$ 1,637.6, and the estimated cost per bed day at primary and secondary hospital level was US$ 16.2 and US$ 67.2, respectively. The average cost by pesticide type across hospital levels was highest for carbamate at US$ 119.9 (5.4–619.2), followed by organophosphate at US$ 107.9 (4.6–653.3). Treatment at the tertiary hospital level accounted for the largest share of the total costs (70.2%). The average cost was US$ 286.6 (20.7–653.3) for treatment at the tertiary level, while treatment costs were US$ 9.7 (2.7–15.5) and US$ 52.1 (7.7–280.3) for primary and secondary level, respectively. See Table 4 for details.

**Table 4.** Average treatment costs by poison type and hospital level for pesticide self-poisoned patients in Anuradhapura District, Sri Lanka, from 1 March to 31 July in 2016.

	Hospital level			
Pesticide type	Primary level (USD, 2015)	Secondary level (USD, 2015)	Tertiary level (USD, 2015)	All hospitals levels (USD, 2015)
Insecticide	10.0 (18)^a^	46.2 (20)	347.8 (10)	95.50 (48)
Carbamate	11.5 (6)	27.9 (5)	341.9 (5)	119.9 (16)
Organosphosphate	9.0 (9)	59.5 (7)	353.7 (5)	107.9 (21)
Other	10.0 (3)	46.0 (8)	- (0)	36.2 (11)
Herbicide	8.7 (7)	66.9 (8)	133.7 (4)	59.5 (19)
All pesticide types	9.7 (25)	52.1 (28)	286.6 (14)	85.3 (67)

^a^Number of patients in parenthesis.

**Figure 1.**
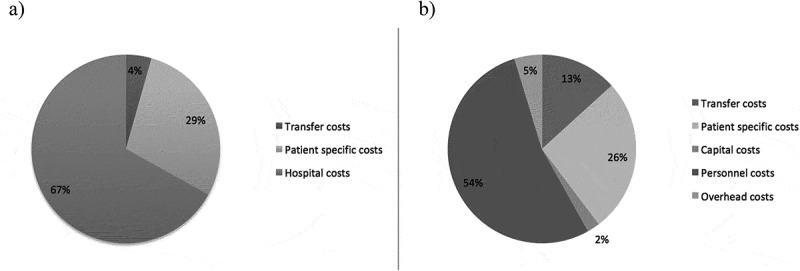
Cost profile. (a) 67 Self-poisoned patients admitted to six different hospitals in the Anuradhapura District from 1 March to 31 July 2016. (b) 53 Self-poisoned patients admitted to five different hospitals in the Anuradhapura District (tertiary level not included) from 1 March to 31 July 2016.

Figure 1(a) shows the distribution of the costs among the components: hospital costs (capital costs, overhead, personnel costs), patient-specific costs (medical prescriptions, medical devices, laboratory tests), and transfer costs (fuel costs). Hospital costs constitute the largest cost component of the total cost for treating self-poisoning cases (67%), and transfer costs the smallest share (4%). Additionally, Figure 1(b) shows a cost profile with a further breakdown of cost components. The profile is based on the five hospitals at the primary and secondary hospital levels since it was not possible to break down tertiary cost data. Hospital costs accounted for 61% of the total costs, of which personnel costs constituted the largest share of expense in the analysis, accounting for 54% of the total costs, followed by patient-specific costs (26%) and transfer costs (13%). A further breakdown of cost components at primary hospital level showed that transfer costs accounted for 47% of the total treatment cost at this level (data not shown).

### Sensitivity analyses of hospital study

The sensitivity analyses showed that the total costs were sensitive to assumptions about personnel (salaries and number of personnel), transfers (fuel prices and number of transfers) and patient-specific costs (medicine and test prices), especially at the primary hospital level. A reduction of 50% in the number of transfers from the primary hospital level to a higher hospital level provided a 25% decrease in costs of treatment at that level. The total cost was not sensitive to changes in discount rates, lifetime of equipment and buildings, construction price of buildings, number and cost of equipment, and overhead. Finally, a sensitivity analysis was carried out on the cost per bed day at the tertiary level obtained from WHO. In a scenario where changes in capital, overhead, and/or personnel costs lead to an increase of 10% in per bed day cost, the total cost increased by almost 5% (data not shown).

### Scenario analyses on the total cost of treating pesticide poisoning cases in Sri Lanka in 2005/2015

Extrapolating our results to the national level, the total cost of treating pesticide poisoning cases (including self-poisoning, accidental, and occupational) in Sri Lanka amounted to US$ 2.5 million or 0.19% of the total government health expenditure (GHE) in Sri Lanka in 2015. The total cost of treating pesticide poisoning cases in the NCP and Anuradhapura District in 2015 was US$ 421,747 and US$ 295,420, respectively, equalling 0.32% and 0.22%, respectively, of the GHE in the NCP.

The results of the scenario analyses on cost and distribution parameters are shown in Table 5. In scenario 1, the total costs of treating all registered pesticide poisoning cases in Sri Lanka increased by 37% between 2005 and 2015. In scenarios 2 and 3, the total cost of treatment of pesticide poisoning cases decreased overall with 20%. However, in all scenarios, the total cost of treatment as a percentage of the total GHE in Sri Lanka decreased over the decade (data not shown).

**Table 5.** Total cost of pesticide poisoning treatment for all patients in Sri Lanka, 2005/2015.

Scenario^a^	Level	Total cost (US$) 2005^b^	Total cost (US$) 2015
Base-case	Sri Lanka	-	2,533,772
	North Central Province	-	421,747
	Anuradhapura District	-	295,420
Scenario 1^c^	Sri Lanka	998,928	1,369,760
	North Central Province	140,801	215,267
	Anuradhapura District	104,886	148,165
Scenario 2^d^	Sri Lanka	864,828	690,723
	North Central Province	136,332	114,971
	Anuradhapura District	97,838	80,533
Scenario 3^e^	Sri Lanka	874,905	695,786
	North Central Province	136,332	115,814
	Anuradhapura District	97,838	81,124

^a^All types of pesticide poisoning (self-poisoning, accidental, and occupational).

^b^Adjusted for inflation.

^c^Registered cases were grouped into two different pesticide categories (organophosphate/carbamate cases, and other pesticide cases) using a ‘distribution key’; the cost of transfer was excluded; and an average cost estimate obtained from this study was used for each pesticide category across all hospital levels.

^d^Registered cases were grouped into two different pesticide categories (organophosphate/carbamate cases, and other pesticide cases) using a ‘distribution key’; the cost of transfer was included; and a cost estimate obtained from this study was used for each pesticide category by hospital level.

^e^Registered cases were grouped into three different pesticide categories (organophosphate, carbamate, and other pesticides) using a ‘distribution key’; the cost of transfer was included; and a cost estimate obtained from this study was used for each pesticide category by hospital level.

## Discussion

The aim of this study was to improve and update the cost estimates of treating pesticide self-poisoning cases in Sri Lanka. The study included 67 patients admitted for pesticide self-poisoning to selected hospitals in the Anuradhapura District over a 4-month study period in 2016. The total cost of pesticide treatment was US$ 5,714, equalling an average cost of US$ 85.3 (9.7–286.6) per patient across all types of pesticide poisoning and hospital levels. In 2015, the total cost of pesticide poisoning treatment to the government was an estimated US$ 2.5 million, or 0.19% of the total GHE.

The most expensive treatment costs per patient were at the tertiary hospital level accounting for 70.2% of the total costs. Relative to other hospitals, tertiary hospitals have high costs because they treat high severity cases transferred from primary and secondary facilities [25].

Our study furthermore found that 58% of the patients admitted to primary and secondary hospitals were transferred to a tertiary hospital, but that transfer costs constituted only 4% of the total costs. However, when breaking cost components down to the primary hospital level, it was found that transfer costs constituted almost half of the total treatment cost for this level. Our sensitivity analyses showed that the treatment cost at this level was sensitive to changes in fuel prices and number of transfers. The high percentage of patients being transferred from lower level hospitals to the teaching hospital (tertiary level) may result from a lack of appropriate equipment, personnel capacity, and available medicines [26]. If investments were made to overcome capacity constraints at lower level hospitals, treatment cost would become higher but sensitivity analyses indicate that investments in these areas would only result in a small increase in total treatment cost. In the case of Bangladesh, Verma et al. [12] argue that investments in peripheral hospitals could affect the number of pesticide cases being transferred to higher hospital levels.

The scenario analyses showed that the total cost of pesticide poisoning treatment as a percentage of the total GHE had decreased over the past decade. This difference may be explained by the increasing cost of other health-care needs, e.g. non-communicable diseases, as well as changes in disease pattern overall, including means of self-harm, types of poisons used for self-harm, and not least the banning of the most hazardous pesticides. Sri Lanka has a long history of restricting access to pesticides through bans, and in the years following the Wickramasinghe study, bans on the most pivotal pesticides in pesticide suicides (paraquat, dimethoate, and fenthion) were implemented making them less available [27–30]. The country then experienced a decline in pesticide suicides following these bans but concurrently saw an increase in other methods to commit suicide [28]. Furthermore, Wickramasinghe et al. [11] found that the cost of treating cases of self-poisoning with highly hazardous pesticides was significantly higher than the cost of treating less hazardous pesticides poisoning cases because they needed care in the ICU.

In the present hospital study, the average length of stay in the hospitals was 26 h and 39 min. This is only one-third of the length of stay estimated in the study by Wickramasinghe et al. [11], suggesting that poisoning cases identified were less severe and required less treatment care. The present study did not identify any patients admitted to the ICU, thereby reducing the length of stay and, thus, the average treatment cost in our sample. As noted above, strict enforcement of bans on highly hazardous pesticides may help to reduce the number of highly costly treatment cases, thereby reducing government expenditures.

Extrapolating our results to the national level the estimated total national treatment cost for pesticide poisoning was more than US$ 2 million in 2015. However, the costs to society exceed this as our estimates are only from a governmental perspective. Suicide is the second leading cause of death among 15–29-year-olds worldwide [31], resulting in huge losses in productivity to society due to their premature deaths. To put this in perspective, Choi et al. [32] estimated the cost of lost productivity to society from pesticide poisoning in South Korea to be 135.9 million, equalling almost 91% of the total cost.

Our cost estimates differed from the previous Sri Lankan study conducted in the same district [11] due to differences in methodology and cost components included in the study. Our average per patient cost of pesticide self-poisoning treatment was higher than that calculated for the Wickramasinghe study from 2005 (US$ 85.3 vs. US$ 48.2 (adjusted for inflation)) due to the inclusion of health-care at the tertiary level. The inclusion of tertiary hospital level in our study also explained the increase in the total cost of pesticide poisoning treatment observed in scenario 1. Differences in hospital types included in the study meant that our average per patient cost at the secondary level was lower than that found in the Wickramasinghe study (base hospital (US$ 52.1) vs. general hospital (US$ 60.1)) [11]. Differences in methodology and country practices also hinder direct comparisons between this and the Bangladeshi study. Our average cost estimate of treating organophosphate poisoning at the tertiary level was found to be higher than the Bangladeshi study (US$ 353.7 vs. US$ 218.1 (adjusted for inflation)) [12]. However, in the latter study capital costs were not included in the calculations of cost per bed day. A further reason why the cost of treatment to the government was higher in Sri Lanka than Bangladesh was due to universal health coverage in Sri Lanka.

### Strengths and limitations

The strength of the hospital study lies in its detailed collection of cost and patient data. A field assistant systematically followed and collected all cost units administered to a patient when admitted for pesticide poisoning at one of the hospitals being monitored. Data were collected from three levels of health-care to estimate the average cost of treating pesticide self-poisoning in Sri Lanka. Furthermore, hospital data pertaining to capital costs, personnel, overhead, and consumables were identified, quantified and ascribed a unit cost.

The hospital study had several limitations. The patient-specific costs are based on a very small sample size of 67 patients. If time and budget had allowed for it, the data collection at each hospital would ideally have continued until a greater sample size of cases had been obtained to ensure a wider representation of types of pesticides and required treatment responses. While poison types identified at primary and secondary level included carbamate insecticides, organophosphorus insecticides, herbicides, and other insecticides, only the three former types of pesticides were identified at the tertiary level. In a study of longer duration, other types of pesticides, including fungicides, may have been identified and included in the costing analysis. Finally, the present study did not identify any patients admitted to ICU care. A longer study period might have resulted in the identification of ICU cases. Another explanation for this lack of finding is that the profile of highly hazardous poisoning cases, and thus, patients in need of ICU, vary across the country. It is likely that the inclusion of more hospitals in different districts would have identified cases admitted to ICU care. It should, therefore, be noted that our cost estimates are conservative and do not reflect the cost of ICU care. The inclusion of ICU costs would inevitably have resulted in higher treatment costs. It must also be noted that due to the short study period, potential seasonal differences in pesticide use may have influenced the types of pesticides found in our survey, making our cost estimations less robust.

Our cost estimates for hospital treatment are conservative. Several assumptions were made in the analysis around operational costs of medical equipment, value and lifetime of buildings, and transfer costs. However, these data limitations were taken into account by means of sensitivity analyses. Furthermore, it was not possible to obtain the necessary cost data to calculate a cost per bed day unit for the tertiary hospital level in Sri Lanka; therefore, the WHO’s estimate was used.

The scenario analyses were based on several assumptions which have affected the estimates for total treatment cost to the Sri Lankan government. Costs of ICU and transfer cases had to be excluded from the scenarios due to lack of available data, meaning that the models underestimated the real cost to the government. The cause of pesticide poisoning was, likewise, unavailable for the registered pesticide poisoned cases in Sri Lanka. The assumption that the cost of treating an occupational poisoned case equalled that of treating a self-poisoned case, however, overestimated the real cost of treatment. Occupational exposure primarily occurs by skin contact or inhalation while spraying pesticides, while pesticide self-poisoning usually happens through ingestion which causes more harm to the system and is, thus, more costly to treat.

Finally, it must be noted that we compared our cost estimates with other studies by adjusting the estimated costs of treatment for inflation. However, there are limitations to this approach as health-care charges vary between countries and over time.

Despite the limitations of the study, our estimates can to an extent indicate which pesticide classes are the most expensive and difficult to treat, and which cost components make up the largest part of treatment work. Costing analyses are sometimes considered of a limited value, but from a preventive perspective, studies such as this provide valuable information that should be taken into consideration in policymaking, budget planning, and assessing investment in potential cost-saving interventions.

## Conclusion

The cost to the government of treating pesticide self-poisoning at different hospital levels was estimated in the present study. The total cost of treatment was estimated to be US$ 5,714 with an average treatment cost of US$ 85.3 (9.7–286.6) per patient. The average cost of treatment was highest at the tertiary level, and hospital costs constituted the largest share of the total cost for treating self-poisoned patients. In the study, we demonstrated that the average per patient cost of pesticide self-poisoning treatment has increased over the past decade. However, an extrapolation of our findings to provincial and national levels suggests that the total cost of treatment of pesticide poisoning as a percentage of the GHE has declined in Sri Lanka over the period. This change is most likely due to national strategies aimed at phasing out the most hazardous and costly to treat pesticides, a shift in methods to commit suicide, and increasing cost of other health-care needs, e.g. non-communicable diseases. A continuous focus on banning the most hazardous pesticides available and enforcing existing bans on highly hazardous pesticides would likely further drive down the cost of pesticide self-poisoning and pesticide poisoning to the government.

## Paper context

Pesticide self-poisoning is a commonly used method of suicide in Sri Lanka, and treatment costs add to the government health expenditures. Costing studies can be used to investigate the cost of illness and the potential for cost-savings. This study contributes to new estimates on treating pesticide self-poisoning cases in Sri Lanka at different hospital levels. Furthermore, the study investigates treatment costs over the past decade in light of Sri Lanka’s regulatory strategies that target the most hazardous pesticides.

## References

[1] World Health Organization Global Health Observatory (GHO) data. 2019 [cited 2019 Nov 21] Available from: http://www.who.int/gho/mental_health/suicide_rates/en/

[2] Knipe DW, Metcalfe C, Gunnell D. WHO suicide statistics - a cautionary tale. Ceylon Med J. 2015;60:35. 25804920 10.4038/cmj.v60i1.7464 PMC4384175

[3] Mew EJ, Padmanathan P, Konradsen F, et al The global burden of fatal self-poisoning with pesticides 2006–15: systematic review. J Affect Disord. 2017;219:93–9. 28535450 10.1016/j.jad.2017.05.002

[4] Gunnell D, Eddleston M, Phillips MR, et al The global distribution of fatal pesticide self-poisoning: systematic review. BMCPublic Health. 2007;7:357. 10.1186/1471-2458-7-357 PMC2262093 18154668

[5] Bertolote JM, Fleischmann A, Eddleston M, et al Deaths from pesticide poisoning: a global response. Br J Psychiatry. 2006;189:201–203. 16946353 10.1192/bjp.bp.105.020834 PMC2493385

[6] Jensen HK, Konradsen F, Jors E, et al Pesticide use and self-reported symptoms of acute pesticide poisoning among aquatic farmers in Phnom Penh, Cambodia. J Toxicol. 2011;2011:639814. 21234245 10.1155/2011/639814 PMC3018643

[7] Gunnell D, Eddleston M Suicide by intentional ingestion of pesticides: a continuing tragedy in developing countries. IntJ Epidemiol. 2003;32:902–909. 14681240 10.1093/ije/dyg307 PMC2001280

[8] United Nations Sri Lanka Free health policy in Sri Lanka. 2016 [cited 2018 830] Available from: http://lk.one.un.org/7060/en/free-health-policy-in-sri-lanka

[9] World Health Organization Humanitarian health action. Sri Lanka. 2019 [cited 2019 48] Available from: http://www.who.int/hac/donorinfo/lka/en/index1.html

[10] Madsen LB, Eddleston M, Hansen KS, et al Cost-effectiveness analyses of self-harm strategies aimed at reducing the mortality of pesticide self-poisonings in Sri Lanka: a study protocol. BMJ Open. 2015;5:e007333. 10.1136/bmjopen-2014-007333 PMC4346671 25724984

[11] Wickramasinghe K, Steele P, Dawson A, et al Cost to government health-care services of treating acute self-poisonings in a rural district in Sri Lanka. Bull World Health Organ. 2009;87:180–185. 19377713 10.2471/BLT.08.051920 PMC2654652

[12] Verma V, Paul S, Ghose A, et al Treatment of self-poisoning at a tertiary-level hospital in Bangladesh: cost to patients and government. Trop Med Int Health. 2017;22:1551–1560. 29064144 10.1111/tmi.12991

[13] Pearson M, Konradsen F, Gunnell D, et al A community-based cluster randomised trial of safe storage to reduce pesticide self-poisoning in rural Sri Lanka: study protocol. BMCPublic Health. 2011;11:879. 10.1186/1471-2458-11-879 PMC3227631 22104027

[14] Pearson M, Metcalfe C, Jayamanne S, et al Effectiveness of household lockable pesticide storage to reduce pesticide self-poisoning in rural Asia: a community-based, cluster-randomised controlled trial. Lancet. 2017;390:1863–1872. 28807536 10.1016/S0140-6736(17)31961-X PMC5655546

[15] Medical Statistics Unit Annual health bulletin 2015. Colombo (Sri Lanka): Ministry of Health, Nutrition and Indigenous Medicine; 2017.

[16] Dalpatadu S, Perera P, Wickramasinghe R, et al Public hospital governance in Sri Lanka. Manila: World Health Organization, Regional Office in the Western Pacific; 2015.

[17] Ministry of Health, Nutrition & Indigeneous Medicine. Health Institution. Human Resource Information of Government Health Sector 2018 [cited 2018 1011] Available from: http://www.health.gov.lk/enWeb/index.php?option=com_content&view=article&id=323&Itemid=137

[18] Gunnell D, Fernando R, Hewagama M, et al The impact of pesticide regulations on suicide in Sri Lanka. IntJ Epidemiol. 2007;36:1235–1242. 17726039 10.1093/ije/dym164 PMC3154644

[19] Drummond MF, Sculpher MJ, Torrance GW, et al Methods for the economic evaluation of health care programmes. New York (NY): Oxford University Press; 2005.

[20] Tan-Torres Edejer T, Baltussen R, Adam T, et al Making choices in health: WHO guide to cost-effectiveness analysis. Geneva: World Health Organization; 2003.

[21] Emmanuel R Estimating the environmental suitability of wall materials: preliminary results from Sri Lanka. Build Environ. 2004;39:1253–1261.

[22] NBM Media Pvt.Ltd Causes for accelerated structural deterioration of reinforced concrete. 2018 [cited 2018 830] Available from: https://www.nbmcw.com/tech-articles/concrete/28072-causes-for-accelerated-structural-deterioration-of-reinforced-concrete.html

[23] World Health Organization CHOosing Interventions that are Cost Effective (WHO-CHOICE). Sri Lanka. 2018 [cited 2018 920] Available from: http://www.who.int/choice/country/lka/cost/en/

[24] Medical Statistics Unit Annual health statistics 2005. Colombo (Sri Lanka): Ministry of Health,Nutrition and Indigenous Medicine; 2007.

[25] Hensher M, Price M, Adomakoh S Referral hospitals In: Jamison DT, Breman JG, Measham AR, et al, editors. Disease control priorities in developing countries. 2nd ed. Washington (DC): The International Bank for Reconstruction and Development/The World Bank; 2006 Chapter 66. p. 1229–1243. Co-published by Oxford University Press, New York. 21250309

[26] Senarathna L, Adams J, De Silva D, et al Personal and professional challenges in the management of deliberate self-poisoning patients in rural Sri Lanka: a qualitative study of rural hospital doctors’ experiences and perceptions. BMCPublic Health. 2008;8:373. 10.1186/1471-2458-8-373 PMC2583998 18954469

[27] Pearson M, Zwi AB, Buckley NA, et al Policymaking ‘under the radar’: a case study of pesticide regulation to prevent intentional poisoning in Sri Lanka. Health Policy Plan. 2015;30:56–67. 24362640 10.1093/heapol/czt096 PMC4287191

[28] Knipe DW, Chang SS, Dawson A, et al Suicide prevention through means restriction: impact of the 2008–2011 pesticide restrictions on suicide in Sri Lanka. PLoS One. 2017;12:e0172893. 28264041 10.1371/journal.pone.0172893 PMC5338785

[29] Knipe DW, Gunnell D, Eddleston M Preventing deaths from pesticide self-poisoning-learning from Sri Lanka’s success. Lancet GlobHealth. 2017;5:e651–e652. 10.1016/S2214-109X(17)30208-5 28619217

[30] Gunnell D, Knipe D, Chang SS, et al Prevention of suicide with regulations aimed at restricting access to highly hazardous pesticides: a systematic review of the international evidence. Lancet GlobHealth. 2017;5:e1026–e1037. 10.1016/S2214-109X(17)30299-1 28807587

[31] World Health Organization Mental health. Suicide data. 2018 [cited 2018 43] Available from: http://www.who.int/mental_health/prevention/suicide/suicideprevent/en/

[32] Choi Y, Kim Y, Ko Y, et al Economic burden of acute pesticide poisoning in South Korea. Trop Med Int Health. 2012;17:1534–1543. 23051841 10.1111/j.1365-3156.2012.03096.x

