# Supplementary material for: Estimating the government health-care costs of treating pesticide poisoned and pesticide self-poisoned patients in Sri Lanka
Source: Glob Health Action. 2019 Nov 28;12(1):1692616. doi: 10.1080/16549716.2019.1692616 (PMC6896413; doi:10.1080/16549716.2019.1692616)
Supplement: Supplemental Material [file ZGHA_A_1692616_SM3971.docx]

**SUPPLEMENTARY SECTION - RESULTS**

**Table 1: Classification scheme of pesticides and their associated chemical group, active compound and hazard classification [1].**

|  |  |  | **Hazard classification** |
| --- | --- | --- | --- |
| **Main use** | **Chemical group** | **Active compound** | **WHO^1^** |
| Insecticide | Carbamate | Carbofuran | Ib^2^ |
|  |  | Carbosulfan | II^3^ |
|  | Organophosphates | Chlorpyrifos | II |
|  |  | Malathion | III^4^ |
|  |  | Profenofos | II |
|  | Pyrethroid | Deltamethrin | II |
|  |  | Etofenprox | U^5^ |
|  | Anthranilic diamide | Chlorantraniliprole | U |
|  | Macrocyclic lactone | Abamectin | n/a^6^ |
|  | Neonicotinoids | Imidacloprid | II |
| Herbicide | Anilide | Propanil | II |
|  | Chloroacetanilide | Pretilachlor | U |
|  | Phenoxyacetic acid derivative/Chlorophenoxy acid or ester | MCPA | II |
|  | Phosphonoglycine | Glyphosinate ammonium | O^7^ |
|  | Pyrimidinyl(thio)benzoate | Bispyribac sodium | III |

^1^ WHO classification based on LD_50_ for rat (mg/kg body weight)

^2^ Highly hazardous

^3^ Moderately hazardous
^4^ Slightly hazardous

^5^ Unlikely to present acute hazard in normal use

^6^ Not included on the WHO and/or GHS classification list of active pesticide ingredients

^7^ Obsolete as pesticide, not classified.

**REFERENCE**

[1] World Health Organization. The WHO Recommended Classification of Pesticides by Hazard and Guidelines to Classification 2009. Geneva: World Health Organization; 2009.
